# Identification of the high affinity binding site in the *Streptococcus intermedius* toxin intermedilysin for its membrane receptor, the human complement regulator CD59

**DOI:** 10.1016/j.molimm.2009.01.003

**Published:** 2009-04

**Authors:** Timothy R. Hughes, Kirsty S. Ross, Graeme J.M. Cowan, Baalasubramanian Sivasankar, Claire L. Harris, Timothy J. Mitchell, B. Paul Morgan

**Affiliations:** a Complement Biology Group, Department of Medical Biochemistry and Immunology, School of Medicine, Cardiff University, Cardiff CF14 4XN, United Kingdom; b Institute of Biomedical and Life Sciences, Division of Infection and Immunity, University of Glasgow, United Kingdom

**Keywords:** C, complement, MAC, membrane attack complex, CDCs, cholesterol-dependent cytolysins, ILY, Intermedilysin, Intermedilysin, CD59, Cholesterol-dependent cytolysins, Complement

## Abstract

The unique species specificity of the bacterial cytolysin intermedilysin is explained by its requirement for the human complement regulator CD59 as the primary receptor. Binding studies using individual domains of intermedilysin mapped the CD59-binding site to domain 4 and swap mutants between human and rabbit (non-intermedilysin-binding) CD59 implicated a short sequence (residues 42–59) in human CD59 in binding intermedilysin. We set out to map more closely the CD59 binding site in intermedilysin. We first looked for regions of homology between domain 4 in intermedilysin and the terminal complement components that bind CD59, C8 and C9. A nine amino acid sequence immediately adjacent the undecapeptide segment in intermedilysin domain 4 matched (5 of 9 identical, 3 of 9 conserved) a sequence in C9. A peptide containing this sequence caused dose-dependent inhibition of intermedilysin-mediated lysis of human erythrocytes and rendered erythrocytes more susceptible to complement lysis. Surface plasmon resonance analysis of intermedilysin binding to immobilized CD59 revealed saturable fast-on, fast-off binding and a calculated affinity of 4.9 nM. Substitution of three residues from the putative binding site caused a 5-fold reduction in lytic potency of intermedilysin and reduced affinity for immobilized CD59 by 2.5-fold. The demonstration that a peptide modeled on the CD59-binding site inhibits intermedilysin-mediated haemolysis leads us to suggest that such peptides might be useful in treating infections caused by intermedilysin-producing bacteria.

## 1 Introduction

The complement (C) system plays a critical role in host defense and is involved in both innate and adaptive immune responses (Walport, 2001). C exerts its various functions directly or indirectly through bioactive cleaved protein fragments (C3a, C5a), or by formation of the cytolytic protein assembly, the membrane attack complex (MAC), formed from the five terminal components C5–C9. Because of the large potential for harm to self-cells posed by unrestricted C activation, stringent control mechanisms have evolved to prevent such damage occurring. Much of this regulation is effected through a battery of control proteins, present both in the fluid phase and on the membranes of our cells (Morgan and Harris, 2003). MAC formation is under the control of CD59, a widely distributed 18–21-kDa (77 amino acids) glycoprotein attached to the plasma membrane by a glycosyl phosphatidylinositol (GPI) anchor (Morgan, 1999; Bodian et al., 1997). CD59 functions by binding to C8 and C9 in the assembling MAC and interfering with the unfolding and membrane insertion of multiple C9 molecules.

The family of structurally related bacterial pore forming toxins known as the cholesterol-dependent cytolysins (CDCs) exhibit several common features (Tweten, 2005). These include, as the name implies, an absolute requirement for cholesterol in the membranes of target cells, a 4-domain structure, and the capacity to oligomerise on target cells to form large trans-membrane pores. In all CDCs, the third domain contains the trans-membrane portion of the protein while domain 4 targets and anchors the toxins to the membrane, initiating large conformational changes allowing oligomerisation and membrane insertion to take place (Rossjohn et al., 2007). A conserved 11-amino acid (undecapeptide) sequence (ECTGLAWEWWR) found in domain 4 of the CDCs was believed to be responsible for binding to cholesterol and insertion into cell membranes (Giddings et al., 2003); however, this dogma has been challenged recently by the demonstration that, while the undecapeptide is required for pre-pore to pore conversion, the L1–L3 loops in domain 4 are primarily responsible for cholesterol binding (Soltani et al., 2007).

Intermedilysin (ILY) is a member of the CDC family produced by *Streptococcus intermedius*. ILY is a major virulence factor for this bacterium; expression is up-regulated up to 10-fold in *S. intermedius* isolates from abscesses and the cytolytic activity of secreted ILY is important in the observed pathology (Nagamune et al., 2000; Sukeno et al., 2005). In several ways, ILY is an unusual member of the CDC toxin family (Nagamune et al., 1996). First, it is uniquely species restricted in its ability to bind and lyse cells, being effective only against human cells. Second, the conserved undecapeptide is altered in ILY with a cysteine, conserved in all other CDCs, being replaced by alanine. Third, while cholesterol is required for initial membrane binding in all other CDCs, ILY binds cells independently of cholesterol, although cholesterol is required for membrane insertion of the oligomerised toxin pore. Interaction of ILY with cells occurred through domain 4 of ILY and was proposed to be mediated by a protein component in the membrane (Nagamune et al., 1996, 2004). Species specificity and cholesterol-independent binding were subsequently explained by Giddings et al. (2004), when they showed that monoclonal antibodies against the human C regulator CD59 blocked the capacity of ILY to bind and lyse human erythrocytes, clearly implicating human CD59 as the specific receptor for ILY. ILY also bound and lysed murine SV-t2 cells expressing human but not rabbit CD59, and binding was mediated through domain 4 of ILY. Swap mutants between human and rabbit CD59 implicated a short sequence in human CD59 (amino acids 42–58) adjacent the site implicated in complement inhibition as the binding site for ILY.

The aim of our current study was to further examine the interaction between ILY and CD59 and identify the residues in ILY responsible for binding CD59. The natural ligand for CD59 in its role as a C regulator is the complement protein C9. There are clear functional similarities between ILY and C9. Both C9 and ILY undergo dramatic conformational changes to oligomerise and form membrane spanning pores, and both bind CD59. Given these observations, we hypothesised that there could be regions of amino acid homology between the two proteins that might underlie these functional similarities. An examination of domain 4 of ILY, which contains the undecapeptide responsible for membrane insertion of the protein, revealed a 9 amino acid sequence that was homologous with human C9. To test the role of this sequence we generated a peptide spanning the homologous region and found that it specifically bound to CD59. Engineered forms of the ILY protein in which this sequence was disrupted showed reduced ability to interact with CD59 and lyse human cells.

## 2 Experimental procedures

### 2.1 Materials

All reagents were from Fisher unless otherwise specified. Human blood was obtained either from the local Blood Transfusion Service or from consenting healthy laboratory volunteers. Chemically competent *Escherichia coli* strains TOP10 and DH5α were purchased from Invitrogen (Paisley, UK), and strains XL1-Blue and BL21 (DE3) were purchased from Stratagene (Amsterdam Zuidoost, The Netherlands).

Recombinant soluble CD59 comprising amino acids 1–76 with an 11 amino acid Ser/Gly linker and terminal Cys residue at the carboxy-terminus was generated both in bacteria and in CHO cells, refolded (for the bacterial product) and purified essentially as previously described (Fraser et al., 2003). CD59 produced in CHO cells was modified to remove the sole *N*-glycosylation site, asparagine 18. The anti-CD59 antibodies HC1 and MEM43 were, respectively, kind gifts of Drs. S. Meri (University of Helsinki, Finland) and V. Horejsi (Academy of Sciences of the Czech Republic, Prague, Czech Republic). Complement fixation diluent (CFD) was from Oxoid (Basingstoke, UK). Phosphate buffered saline (PBS) was 10 mM phosphate pH 7.2, 160 mM NaCl.

HEPES-buffered saline was 10 mM HEPES pH 7.4, 150 mM NaCl. All primers were obtained desalted from Sigma-Genosys Ltd. (Gillingham, UK). Primers for site-directed mutagenesis were further purified by polyacrylamide gel electrophoresis (PAGE) before use. All enzymes used for DNA manipulations and ligations were obtained from Promega (Southampton, UK) except for Pcil, which was obtained from New England Biolabs (Hitchin, UK). Peptide synthesis was performed in-house on an ABI 433A peptide synthesiser (Applied Biosystems Ltd., Warrington, UK). Both peptides were insoluble in water and DMSO and were initially dissolved in 30% acetic acid or DMF before dilution in aqueous buffer to the concentrations described in the figures.

### 2.2 Generation of Fab fragments

Fab fragments of HC1 and MEM43 were prepared by papain digestion. Purified IgG (5 mg/ml) was dialysed against 100 mM sodium phosphate buffer, pH 7.0. Cysteine–HCl (1 mg/10 mg IgG), EDTA (0.5 mg/10 mg IgG) and papain (1 mg/25 mg IgG) were added to the dialysed IgG and the mixture incubated overnight at 37 °C. Residual intact IgG and Fc fragments were removed by passage of the digest through a ProsepA column equilibrated in PBS and the Fab fragments were finally purified by gel filtration on a Superdex 200 FPLC column equilibrated in PBS.

### 2.3 Generation of recombinant ILY and ILY mutants

The coding sequence for mature ILY (mILY) was amplified by PCR from the plasmid pQE9mILY (gift of Prof. Nagamune, University of Tokushima) (Nagamune et al., 1996). Primers were selected to amplify the coding sequence without the putative signal sequence (Table 1: primers 1 and 2). The amplified fragment was gel purified, digested with BamHI and SacI and ligated into a pET33b plasmid (Merck, Nottingham, UK) to facilitate high-level bacterial expression with the addition of a carboxy-terminal six-His tag for purification. The plasmid was transformed into XL1-Blue *E. coli*, positive colonies selected and validated by PCR flanking the insert. Validated plasmid was transformed into BL-21(DE3) *E. coli* (Stratagene) for protein over-expression.

Selected colonies were grown to mid-log phase, IPTG (1 mM) was added to induce expression and cultures incubated for between 3 and 18 h at 37 °C. Bacteria were harvested by centrifugation, pellets re-suspended in PBS containing DNAse I and Benzamidine (Sigma, Southampton, UK). Bacteria were then lysed either by sonication or in a One-Shot Cell Disruptor (Constant Systems, Daventry, UK). Bacterial lysates were centrifuged at 18,000 × *g* and 4 °C for 30 min and the supernatant filtered (0.2 μm) to remove residual cell debris.

To purify the His-tagged protein, cell-free supernatant was applied to a nickel-charged NTA column (Superflow; Qiagen, Crawley, UK). Bound protein was eluted in a 0–300 mM continuous imidazole gradient in PBS, dialysed three times against a 50-fold excess volume of PBS and concentrated in an Amicon Centrifugal Concentrator with 50 kDa molecular weight cut-off (Millipore, Watford, UK). Proteins were further purified by anion exchange (HQ) on a BioCad 700E Workstation (Applied Biosystems Ltd.). DNA and other contaminants were retained on the column and purified proteins present in the flow-through were immediately dialysed and concentrated as above. All proteins were run on SDS-PAGE, Coomassie-stained and protein purity estimated. Absorbance scans from 220 to 320 nm were performed on purified proteins using a Unicam UV2 spectrophotometer to confirm absence of contaminating DNA/RNA.

A fluorescent recombinant form of ILY (designated: Δ6eGFPsILY) was engineered which retained its ability to bind to Human CD59 while having no lytic function. The vector coding for this form of ILY was constructed as follows.

eGFP was amplified from pET33bEGFP-PLY using the primers 3 and 4 (Table 1). ILY was amplified from pET33bILY using primers 5 and 6 (Table 1). PCR products were gel purified and an overlap PCR was performed using primers 7 and 8 (Table 1) to join the two products. The product was digested with SpeI and SacI, gel purified and ligated into pET33b, which had been cut with NheI/SacI and CIAP treated (Merck, Nottingham, UK). The ligation reaction was transformed into TOP10 *E. coli*. Finally, to remove lytic function, a Δ6 mutation (deletion of residues equivalent to A204 and R205 of ILY precursor) was introduced into the pET33bEGFPsILY by site-directed mutagenesis (Quikchange SDM kit, Stratagene), using primers 9 and 10 (Table 1).

Two swap mutations were introduced into the putative CD59-binding site in ILY by site-directed mutagenesis (Quikchange SDM kit, Stratagene); SKN > NRT and LI > TV. The SKN > NRT switch required a two-stage process using primers 11 and 12 followed by primers 13 and 14 (Table 1). The LI to TV switch was carried out in one step using the primers 15 and 16 (Table 1). The introduced changes were confirmed by DNA sequencing on an ABI 310 sequencer (University of Glasgow Molecular Biology Support Service). The mutated proteins were expressed and purified as described above.

### 2.4 Hemolytic assays

Hemolytic assays for ILY activity were performed using human erythrocytes by modification of a published technique (Walker et al., 1987). In brief, 50 μl of a 2% suspension of erythrocytes in PBS was incubated at room temperature with an equal volume of dilutions of the toxin under test for 5–15 min. Plates were centrifuged at 1000 × *g*, supernatants harvested and the absorbance at 540 nm measured in a Dynex Revelation microplate reader (Dynex, Worthing, UK). Percentage lysis was calculated using 100% (water) and 0% lysis controls. Log_10_ toxin concentration was plotted against %hemolysis and the amount of toxin causing 50% lysis (1 hemolytic unit; HU) calculated. Specific activity of a particular toxin was expressed as HU/mg protein.

Hemolytic assays for C activity were performed using human erythrocytes sensitised with an in-house rabbit polyclonal antiserum against human CD55 as described (Cole et al., 2006). Sensitised erythrocytes (2% in CFD; 50 μl/well) were incubated for 30 min with various dilutions of human serum in CFD in the absence or presence of test peptides or blocking anti-CD59 antibodies. Plates were centrifuged and percent lysis calculated as above. Statistical analyses were carried out using unpaired Student's *t*-tests.

### 2.5 ELISA

Microtiter plates (96-well Nunc MediSorp, Life Technologies, Paisley, UK) were directly coated with soluble CD59 (N18Q glycosylation mutant 10 μg/ml in ELISA coating buffer) at 37 °C for 1 or 16 h at 4 °C. Wells were then blocked by incubation with 5% (w/v) milk in PBS. After further washing, peptides (ILY-derived or jumbled control) at various dilutions in PBS were added to the plates. Following a 15 min incubation at 37 °C the peptides were removed without washing and recombinant ILY [4 μM] was added to every well which had received peptides. The plates were incubated for 5 min at 37 °C. Binding of ILY was then detected using an in-house rabbit polyclonal anti-ILY antiserum diluted 1/2000 in PBS, followed by a peroxidase-conjugated donkey anti-rabbit IgG (Jacksons; 1:5000 in PBS). Incubations were performed at 37 °C for 1 h unless otherwise indicated. Between each step, except between peptide and ILY incubations, the wells were washed extensively with PBS containing 0.1% (v/v) Tween-20 (PBS-T). Wells coated only with CD59 served as negative controls. Bound peroxidase was detected with OPD chromogenic substrate (Dako, Cambridgeshire, UK) and absorbance read at 492 nm. All the experiments testing for blocking of binding of ILY to CD59 were repeated at least twice in triplicate.

### 2.6 Flow cytometry

Δ6eGFPsILY at a final concentration of 65 ng/ml (0.8 nM) was added to human erythrocytes (0.02%, v/v) in PBS + 1% BSA in the presence or absence of excess Fab fragments of anti-CD59 antibodies MEM43 or HC1. After 15 min incubation at room temperature the cells were acquired on a flow cytometer (FACSCalibur; BD Biosciences) with settings adjusted for human erythrocytes. Sheep erythrocytes were used as a control for the specificity of Δ6eGFPsILY binding.

### 2.7 Biosensor analysis

All surface plasmon resonance (SPR) analyses were carried out on a Biacore T100 (Biacore International SA, Stevenage, UK). Proteins were coupled to the sensor chip surface using thiol-based coupling chemistry. Briefly, recombinant CD59 was subjected to mild reduction for 16 h at 20 °C using TCEP at a molar ratio TCEP:CD59 of 3:1 [120 μm:40 μM] to reduce the terminal Cys residue while leaving internal disulphide bonds intact. An S-series CM5 carboxy-dextran coated chip was then activated using NHS/EDC (*N*-hydroxysuccinimide/1-ethyl-3(3-dimethyl aminopropyl)-carbodiimide hydrochloride) according to the manufacturer's protocol. The activated surface was further modified by flowing 80 mM 2-(2-pyridinyldithio)-ethaneamine hydrochloride (PDEA) in 0.1 M sodium borate (pH 8.5) to introduce reactive disulphide groups into the matrix. Reduced CD59 was flowed over this surface at pH 3.4 (10 mM NaCitrate) and coupling through the free amino-terminal thiol in the reduced CD59 monitored in real time. Blocking of residual activated residues was achieved by flowing 50 mM l-cysteine in 100 mM NaAcetate pH 4 containing 1 M NaCl. The CD59-coated surface so formed was stable through multiple regenerations. Native conformation of the immobilized CD59 was confirmed by showing efficient binding of two conformation-specific monoclonal anti-CD59 antibodies, HC1 and MEM43 (not shown). For all kinetic analyses, data were collected at 25 °C at a flow rate of 30 μl/min. Data from a reference cell subjected to derivitisation and blocking but without CD59 were subtracted to control for bulk refractive index changes. The maximum binding (*R*_max_) was kept low and the flow rate high to eliminate mass transfer effects. Interactions were analysed in HBS containing 0.05% surfactant P20 and 3 mM EDTA. Data were evaluated using Biacore T100 Evaluation software. Concentration of analytes was assessed immediately prior to kinetic analyses by determining the absorbance at 280 nm (for ILY an absorbance reading of 1 corresponds to a concentration of 0.9 mg/ml).

## 3 Results

### 3.1 A peptide derived from a C9 homology region in ILY inhibits ILY-induced lysis of human erythrocytes

**Fig. 1:**
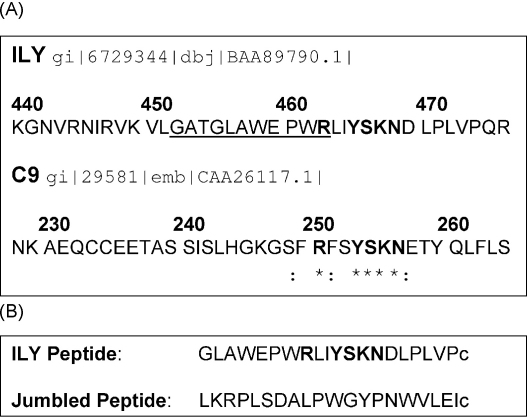
Comparison of protein sequences. (A) Alignment of sequences of ILY and C9 (the natural ligand for CD59) revealed a region of homology residing between amino acids 249 and 257 of C9 and amino acids 461 and 469 of ILY (shown in bold). The underlined portion of the ILY sequence adjacent to the region of homology represents the undecapeptide responsible for membrane binding of the toxin. Asterisk (*) denotes identical residues, (:) denote conservative substitutions. (B) Sequences of the ILY peptide (with homology in bold) and its jumbled control derived from the region of C9 homology. Cysteine residues (shown in lower case) were added at the C-terminal end of each peptide.

**Fig. 2:**
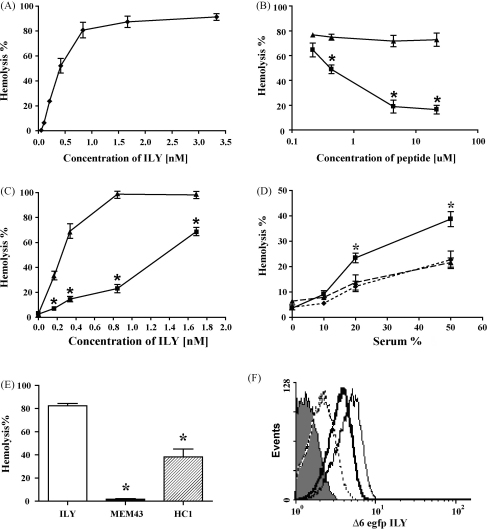
ILY peptide inhibits the interaction of ILY with CD59. (A) Dose-dependent hemolysis of human erythrocytes by ILY. (B) ILY peptide (■) or its jumbled control (▴) were incubated with human erythrocytes in the presence of a constant dose of ILY (40 ng/ml; 0.676 nM) (*N* = 3, ^*^P < 0.05). (C) ILY mediated hemolysis of human erythrocytes in the presence of constant concentrations of ILY peptide (50 μg/ml; 21.7 μM; ■), or its jumbled control (▴) (*N* = 3, ^*^P < 0.05). (D) The effects of ILY-peptide competition on hemolysis of human erythrocytes by complement. Human erythrocytes were incubated with anti-DAF polyclonal antibody, followed by either buffer alone (♦), ILY peptide (■), or jumbled peptide (▴), each at 50 μg/ml (21.7 μM). Antibody-coated erythrocytes were then exposed to normal human serum at the indicated doses. The asterisks indicate significant differences between ILY peptide and the controls (*P* < 0.05, *N* = 3). (E) Human erythrocytes were pre-incubated with Fab fragments (75 μg/ml) of the anti-CD59 monoclonal antibodies MEM43 or HC1 followed by ILY (0.8 nM) (*N* = 3, ^*^P < 0.05 ILY vs ILY plus MEM43 or ILY plus HC1). (F) Binding of the fluorescent, non-lytic ILY fusion protein, Δ6eGFPsILY (0.8 nM) to human erythrocytes as assessed by flow cytometry in the presence or absence of Fab fragments of MEM43 and HC1. The light, solid line shows binding of Δ6eGFPsILY in the absence of Fab; the heavy, solid line shows binding in the presence of HC1(Fab); the broken line shows binding in the presence of MEM43 Fab; the shaded profile is binding of Δ6eGFPsILY to sheep erythrocytes, included as a negative control.

An alignment of the amino acid sequences of ILY (gi|6729344|dbj|BAA89790.1|) and C9 (gi|29581|emb|CAA26117.1|) revealed a short stretch of high homology between residues 249–257 in C9 and residues 461–469 in ILY (Fig. 1). A peptide containing this ILY sequence with flanking residues was synthesised in order to test its capacity to bind CD59 (Fig. 1). A jumbled peptide containing the same amino acids was also synthesised as a control. In the absence of peptide, ILY caused efficient lysis of human erythrocytes, with 100% lysis of a 2% suspension occurring at 1 nM concentration of toxin (Fig. 2A). When tested in human erythrocyte hemolysis assays, the “sense” peptide caused a dose-dependent inhibition of ILY-mediated hemolysis (Fig. 2B). The jumbled peptide caused no inhibition (Fig. 2B). Titration of the dose of ILY [10–100 ng/ml:0.169–1.69 nM] with a constant concentration of peptide [50 μg/ml; 21.7 μM] showed that 5-fold more ILY was needed to cause 50% hemolysis in the presence of the ILY peptide compared to either no peptide or jumbled peptide (Fig. 2C). To test the effect of the peptide on the capacity of CD59 to inhibit C lysis, antibody-sensitised human erythrocytes were pre-incubated with the “sense” or jumbled peptides [50 μg/ml; 21.7 μM] and then exposed, without washing, to human serum. In the presence of the “sense” peptide, C-mediated hemolysis was enhanced, suggesting that the peptide inhibited the C inhibitory activity of CD59 (Fig. 2D). By contrast, the jumbled peptide did not enhance C lysis. At the dilutions used in these assays, neither “sense” nor jumbled peptide caused any lysis above background. When CD59 was neutralised with a blocking monoclonal antibody (MEM43), C-mediated hemolysis was enhanced as predicted; however, the “sense” peptide at concentrations that enhanced hemolysis of unmodified erythrocytes, did not further enhance C-mediated hemolysis of CD59-neutralised erythrocytes, confirming that the peptide affected CD59 function (data not shown). Effects of the monoclonal antibodies on ILY-mediated lysis were also tested. Complete inhibition of ILY-mediated hemolysis was achieved by the addition of a Fab fragment of MEM43, an antibody which binds to the C inhibitory site of CD59 (Fig. 2E), while a Fab fragment of HC1, binding an epitope on CD59 remote from the complement inhibitory site, caused much less inhibition of ILY mediated hemolysis (Fig. 2E). These results were confirmed using a fluorescent, non-lytic, engineered version of ILY (Δ6eGFPsILY). Preincubation of human erythrocytes with Fab fragments of MEM43 prior to Δ6eGFPsILY produced a 5-fold decrease in the fluorescence observed when erythrocytes were incubated with Δ6eGFPsILY alone (Fig. 2F); by contrast, Fab fragments of HC1 caused less than 2-fold decrease in Δ6eGFPsILY fluorescence.

### 3.2 The C9-homologous ILY peptide binds specifically to immobilised CD59

**Fig. 3:**
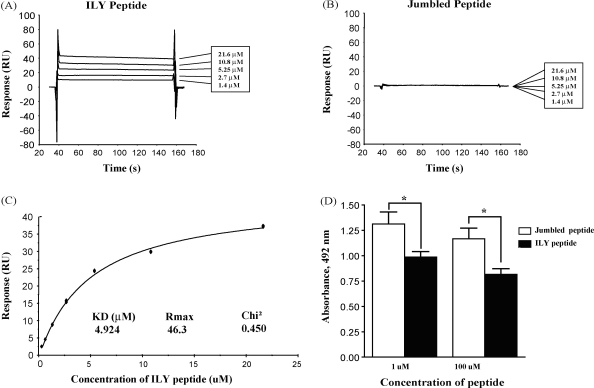
Direct demonstration of interaction between the ILY peptide and CD59. CD59 was immobilized on a CM5-S chip surface via thiol-coupling. Interaction with the ILY-derived peptide (A) or jumbled peptide (B) was analysed at the indicated concentrations in HBS-P. Spikes at the beginning and end of the injection period in (A) are buffer artefacts that do not influence analysis of the data. (C) The affinity of the ILY peptide for CD59 was determined at steady state by flowing peptide over immobilised CD59 at a range of concentrations; the data were analysed using BIAcore T100 evaluation software to obtain the affinity of the ILY peptide for CD59. (D) ELISA plates coated with CD59 were incubated in buffer alone or with either the ILY-derived peptide (solid bars) or its jumbled control (open bars) at 1 and 100 μM final concentration. The peptides were removed and ILY (4 μM) was added for 5 min at 37 °C. After washing, binding of ILY to immobilised CD59 was detected with a polyclonal anti-ILY antiserum. Each bar is the mean of quadruplicate wells. Asterisks denote significant differences (*P* < 0.05).

**Fig. 4:**
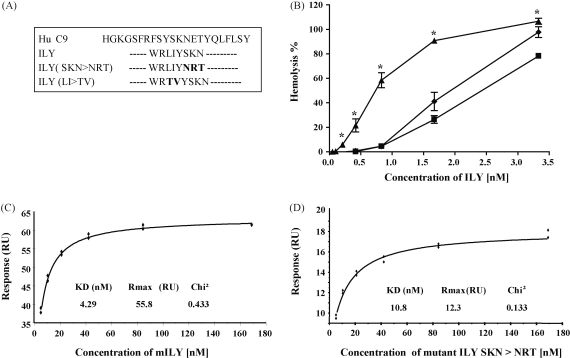
Mutation of the putative CD59 interacting region in ILY confirms functional significance. (A) Two mutants of ILY were prepared, each with altered amino acids in the putative CD59 binding region of ILY. (B) Comparison of the Hemolytic capacity of the ILY mutants (SKN > NRT (■); LI > TV (♦)) with wild type ILY (▴). The asterisk denotes significant differences compared to wild type (*P* < 0.05, *N* = 3). (C) The affinity of wild type ILY for immobilised CD59 was measured by SPR at steady state in the concentration range 169–5.25 nM; the calculated affinity was 4.29 × 10^−9^ M. (D) When the SKN > NRT mutant was similarly analysed, the calculated affinity was 1.08 × 10^−8^ M, a 4-fold decrease.

To confirm that the ILY-derived peptide directly bound CD59, we examined by SPR its interaction with CD59 immobilised through its carboxy terminus to dextran on a Biacore chip. First, a concentration series of the ILY peptide and its jumbled control were flowed over the CD59 chip; the ILY peptide bound specifically but with very fast on/off rates (association/dissociation), whereas the jumbled control peptide did not bind (Fig. 3A and B). The affinity of the ILY peptide for CD59 was determined by steady state analysis of data obtained from a peptide concentration series run in duplicate over the CD59 coated chip (Fig. 3C). The measured affinity was 4.92 × 10^−6^ M with a Chi^2^ value of 0.45. By comparison, the affinity of native ILY for CD59, measured in the same way, was 4.29 × 10^−9^ M, a three-log higher affinity (Fig. 4C). The integrity of the CD59 immobilised on the chip surface was confirmed before and after passage of the peptides by flowing the anti-CD59 monoclonal antibody MEM43, which bound strongly and comparably before and after peptide analysis (data not shown). When interactions were tested in the reverse orientation, with peptides coupled to the chip surface through their terminal cysteine residues and flowing CD59 over the coated surface, no specific interaction was observed despite efficient coupling of peptide to the chip surface (negative data not shown). We concluded that the interaction was hindered by a combination of small peptide size and tethering to a fixed surface, impeding its ability to interact with its binding site on CD59 (data not shown). To provide further confirmation of the specificity of this interaction we examined the capacity of the ILY-derived peptide to directly inhibit the binding of ILY to immobilised CD59 in an ELISA assay. The ILY-derived peptide, but not its jumbled control, significantly inhibited the binding of ILY (Fig. 3D). Poor solubility of the peptides limited our ability to examine the effects of higher amounts of peptides in these competition assays.

### 3.3 Mutating conserved residues in the C9-homologous region of ILY causes reduced hemolytic capacity and diminished affinity for CD59

To confirm the functional importance of the region of C9 homology identified in ILY, we attempted to ablate the region in ILY by targeting common residues in the ILY/C9 homology stretch by site-directed mutagenesis; residues were altered to those present at that position in pneumolysis. Two mutant proteins were successfully generated and expressed, the first swapping LI > TV and the second SKN > NRT in this region (Fig. 4A). These proteins were purified to homogeneity and tested in haemolytic assays. In comparison with native ILY, the hemolytic activity of the SKN > NRT mutant was dramatically impaired, the dose required to cause 50% hemolysis increasing over 3-fold (Fig. 4B). The LI > TV mutant also showed impaired ILY function, although to a slightly lesser extent, a 2.5-fold increase in dose required for 50% hemolysis (Fig. 4B). SPR analyses of native ILY and the SKN > NRT mutant were then carried out to determine the affinities of each toxin for CD59. Immediately prior to each kinetic experiment, the toxin preparations were ‘polished’ on a superdex 200 FPLC gel filtration column to remove any aggregates or breakdown products and equilibrate in Biacore HBS running buffer. Steady state analyses of the data demonstrated that native ILY bound CD59 with a high affinity (KD 4.29 × 10^−9^ M) (Fig. 4C). The affinity of the SKN > NRT mutant was reduced 2.5-fold (KD 1.08 × 10^−8^ M), supporting the hemolysis data and showing that this change reduced but did not ablate the capacity of ILY to bind CD59. The LI > TV mutant was not tested by SPR because of poor solubility in Biacore buffer at the high concentrations needed for analysis.

## 4 Discussion

ILY, a CDC toxin secreted by *S. intermedius*, stands out from the other toxins of this family principally because it lyses only human cells (Nagamune et al., 1996). This is because it utilises human CD59 (and no other species CD59 analogues) as its ligand (Giddings et al., 2004). The binding site in ILY for CD59 had already been localised to domain 4, which shows the same species-specific cell binding properties as intact ILY (Nagamune et al., 2004). We therefore examined domain 4 for clues as to where the CD59 binding site might lie and based on the clear structural and functional similarities between ILY and C9 we looked for regions of amino acid homology between the two proteins. We found a nine amino acid hydrophilic sequence immediately distal to the hydrophobic undecapeptide in domain 4 of ILY that was homologous with a region in C9 (residues 249–257), with five residues identical and three conservative substitutions. We also compared the amino acid sequence of ILY with C8, also involved in the interaction of the forming C5b-9 complex with its regulator CD59. However, in this case we could find no regions of homology between the two proteins.

A synthetic peptide containing this sequence inhibited ILY-mediated hemolysis of human erythrocytes, suggesting that it competitively inhibited binding of ILY to CD59 on erythrocytes. A jumbled from of the peptide had no effect on hemolysis. Importantly, inhibition of hemolysis required a 100-fold molar excess of peptide over ILY and significant inhibition of hemolysis was only seen when the peptide was present during exposure to ILY. When erythrocytes were pre-incubated with peptide and washed prior to exposure to ILY, little or no inhibition of hemolysis was seen. These findings suggest that the peptide had a low affinity for CD59 compared to native ILY. This was confirmed by SPR analysis of binding of ILY and the inhibitory peptide to CD59; ILY bound with a very high affinity (nanomolar) whereas the peptide showed specific but very low affinity (micromolar) binding to CD59 with rapid association and dissociation rates. Jumbled peptide did not bind, confirming specificity. These binding data were confirmed in ELISA, where the ILY-derived peptide but not its scrambled control inhibited the binding of ILY to CD59. The low affinity of peptide for CD59 explains the requirement for a large molar excess of peptide and the absence of inhibition following washing. The thousand-fold higher affinity of native ILY for CD59 compared to peptide likely represents a requirement for a rigid structure around the binding site for high affinity interaction with CD59. An alternative explanation is that there are additional binding domains for CD59 in ILY. We cannot rule this out but, given that the isolated domain 4 had an affinity for CD59 that was near identical to that of native ILY (our unpublished data), it is unlikely that sites outside of this domain contribute to CD59 binding.

The ILY peptide also inhibited the capacity of CD59 to protect cells from C-induced lysis, strongly suggesting that the ILY peptide (and by inference, ILY itself) binds in or close to the “active site” for complement regulation identified in a hydrophobic groove on the membrane-distal face of CD59 (Bodian et al., 1997; Morgan and Tomlinson, 2005). This interpretation is compatible with the observation that a monoclonal antibody directed against an epitope including Arg53 in the C regulatory “active site” of CD59 blocked binding of ILY to CD59 (Giddings et al., 2004). We have independently confirmed this finding using Fab fragments of two different monoclonal antibodies mapped on CD59; only the mAb MEM43, binding close to the “active site”, efficiently blocked ILY interaction with CD59 (Fig. 2E and F). A minor effect on ILY binding and lysis caused by HC1(Fab), a mAb which binds remotely from the “active site” of CD59, was likely due to steric hindrance. This result is compatible with the data of Giddings et al. (2004) who also found some reduction in the binding and haemolysis caused by ILY in the presence of an antibody whose binding site was on the opposite face of CD59 to the “active site” of the molecule.

To confirm that the identified region in ILY was important in the CD59/ILY interaction we mutated the core homologous residues (LI > TV; SKN > NRT) present in the ILY protein. The residues chosen to replace those present in the peptide were taken from the equivalent sequence in a non-CD59 binding but structurally related CDC (human platelet aggregation factor, from *Streptococcus mitis*). Hemolysis assays using these mutated forms of ILY showed that the hemolytic capacity of each of the mutated toxins was markedly reduced compared to the native protein. SPR analysis demonstrated that introduction of the SKN > NRT switch reduced the affinity of ILY for CD59 by 2.5-fold. These data confirm a role for this ILY site in binding CD59, but do not eliminate the possibility that other sites contribute in the intact molecule.

The observations reported here are of obvious relevance for treating infections with ILY-producing organisms. Peptides similar to the one described here, optimised for inhibition of ILY binding to CD59, applied locally or systemically, might reduce cell lysis and consequent pathology. The fact that such agents would also block the complement regulatory activity of CD59 would need to be considered but it is likely that this would not be a major problem in short-term administration. An alternative strategy that would preserve CD59 complement inhibiting activity would be to identify the ILY-binding residues in CD59 and design peptides from this information that bind ILY and prevent binding to CD59.

## Figures and Tables

**Table 1:** List of primers used.

Primer 1	5′-CGG GAT CCG GAA ACA CCT ACC AAA CCA AAA GCA-3
Primer 2	5′-GAC GGA GCT CGA TTA ATC AGT GTT ATC TTT CAC-3′
Primer 3	5′-GTC AGA CTA GTA TGA GTA AAG GAG AAG AAC-3′
Primer 4	5′-GAG CTT TTT TTG CCG GAT CTT TGT ATA GTT CAT C-3′
Primer 5	5′-GAC GGA GCT CGA TTA ATC AGT GTT ATC TTT CAC-3′
Primer 6	5′-GAA CTA TAC AAA GAT CCG GCA AAA AAA GCT CTG AAT G-3′
Primer 7	5′-GTC AGA CTA GTA TGA GTA AAG GAG AAG AAC-3′
Primer 8	5′-GAC GGA GCT CGA TTA ATC AGT GTT ATC TTT CAC-3′
Primer 9	5′-CTA AAA CTC ATG CTG TAC CAA TGC AAT ATG AAT CTA TTA GC-3′
Primer 10	5′-GCT AAT AGA TTC ATA TTG CAT TGG TAC AGC ATG AGT TTT AG-3′
Primer 11	5′-GAC TGA TCT ATA GCA AGA CCG ATC TTC CTT TGG TTC C-3′
Primer 12	5′-GGA ACC AAA GGA AGA TCG GTC TTG CTA TAG ATC AGT C-3′
Primer 13	5′-GCC TTG GAG ACT GAT CTA TAA CCG GAC CGA TCT TCC TTT GGT TC-3′
Primer 14	5′-GAA CCA AAG GAA GAT CGG TCC GGT TAT AGA TCA GTC TCC AAG GC-3′
Primer 15	5′-GAG CCT TGG AGA ACG GTT TAT AGC AAG AAC-3′
Primer 16	5′-GTT CTT GCT ATA AAC CGT TCT CCA AGG CTC-3′
